# A hybrid next generation transcript sequencing-based approach to identify allelic and homeolog-specific single nucleotide polymorphisms in allotetraploid white clover

**DOI:** 10.1186/1471-2164-14-100

**Published:** 2013-02-13

**Authors:** Istvan Nagy, Susanne Barth, Jeanne Mehenni-Ciz, Michael T Abberton, Dan Milbourne

**Affiliations:** 1 Crops, Environment and Land Use Programme, Teagasc, Oak Park, Carlow, Ireland; 2 Current address: Department of Molecular Biology and Genetics, Aarhus University, Forsøgsvej 1, Slagelse, DK-4200, Denmark; 3 Institute of Biological, Environmental & Rural Sciences, Aberystwyth University Penglais, Penglais, Aberystwyth, Ceredigion, SY23 3DA, UK; 4 Current address: International Institute of Tropical Agriculture, PMB 5320, Oyo Road, Ibadan, Nigeria

**Keywords:** Allelic variants, Allotetraploid, Haplotype reconstruction, Next generation sequencing, Single nucleotide polymorphisms, Sub-genome, Transcript assembly, Trifolium, White clover

## Abstract

**Background:**

White clover (*Trifolium repens* L.) is an allotetraploid species possessing two highly collinear ancestral sub-genomes. The apparent existence of highly similar homeolog copies for the majority of genes in white clover is problematic for the development of genome-based resources in the species. This is especially true for the development of genetic markers based on single nucleotide polymorphisms (SNPs), since it is difficult to distinguish between homeolog-specific and allelic variants. Robust methods for categorising single nucleotide variants as allelic or homeolog-specific in large transcript datasets are required. We illustrate one potential approach in this study.

**Results:**

We used 454-pyrosequencing sequencing to generate ~760,000 transcript sequences from an 8th generation white clover inbred line. These were assembled and partially annotated to yield a reference transcript set comprising 71,545 sequences. We subsequently performed Illumina sequencing on three further white clover samples, generating 14 million transcript reads from a mixed sample comprising 24 divergent white clover genotypes, and 50 million reads on two further eighth generation white clover inbred lines. Mapping these reads to the reference transcript set allowed us to develop a significant SNP resource for white clover, and to partition the SNPs from the inbred lines into categories reflecting allelic or homeolog-specific variation. The potential for using haplotype reconstruction and progenitor genome comparison to assign haplotypes to specific ancestral sub-genomes of white clover is demonstrated for sequences corresponding to genes encoding dehydration responsive element binding protein and acyl-coA oxidase.

**Conclusions:**

In total, 208,854 independent SNPs in 31,715 reference sequences were discovered, approximately three quarters of which were categorised as representing allelic or homeolog-specific variation using two inbred lines. This represents a significant resource for white clover genomics and genetics studies. We discuss the potential to extend the analysis to identify a “core set” of ancestrally derived homeolog specific variants in white clover.

## Background

White clover (*Trifolium repens* L.) is an allotetraploid forage legume with a genetically determined gametophytic self incompatibility system [1]. This reproductive feature renders it an obligate outbreeder, resulting in high levels of inter- and intragenotypic heterozygosity. *T. occidentale* D.E. Coombe, and *T. pallescens* Schreber are the currently extant species that seem to best represent the diploid progenitors of the species [2], although more recent analyses [3] seem to suggest that, while the former is a good candidate, the latter exhibits a greater divergence from the sub-genome to which it has been ascribed. Regardless of the identity of the progenitors, the earliest genetic mapping experiments revealed that the two constituent sub-genomes of white clover exhibit highly conserved macrosynteny, with eight homeolog pairs of linkage groups [4,5].

This highly conserved type of allopolyploidy, combined with high intragenotypic heterozygosity, is problematic for the development of sequence-based resources for white clover. This is because the presence of two meiotically independent, but very similar genomes results in a much reduced ability to distinguish between homolog and homeolog copies of genes. The earliest SNP discovery and validation studies in white clover represent a good illustration of the problems associated with distinguishing these two types of variation. Sawbridge et al. [6] generated a collection of over 40,000 expressed sequence tags (ESTs) that were clustered to generate a non-redundant set of over 14,000 consensus sequences. These were subsequently utilised for SNP discovery, exploiting the fact that the underlying cDNA libraries were constructed in multiple, heterogeneous genotypes of the white clover variety Grasslands Huia [7]. White clover varieties are generated via polycrosses of multiple parents and thus are relatively genetically heterogeneous (Grasslands Huia is a synthetic variety derived from seven individual parents [8]) leading to an expectation of multiple allelic variants for individual loci in the consensus sequences generated by clustering. From the EST data, a total of 18,517 candidate SNPs were identified across 1409 loci [9]. On validating SNPs from a small subset of these loci in mapping populations, almost half of the SNP assays generated monomorphic patterns in F_1_ progeny despite prior validation as polymorphic markers [10]. The authors attributed this to the frequent clustering of sequences from highly similar homeolog copies of genes in the consensus sequences generated in the earlier part of the process, and a subsequent inability to efficiently distinguish between allelic and homeolog specific variation amongst the clustered sequences when designing assays. Basing SNP assays on homeolog specific, rather than allelic variants, results in monomorphic assays because alternative homeolog variants are ubiquitously present in segregating populations.

One potential approach for distinguishing homologous, homeologous (and paralagous) sequence variation in EST-derived sequences from outbreeders is the generation, cloning and sequencing of PCR amplicons based on the ESTs. This approach, which allows unambiguous reconstruction of the component haplotypes at a locus (or closely related homeo-loci) was examined by Hand et al. [11]. For four mapping parents, a sufficient number of PCR amplicons were sequenced to represent all forms of variation associated with over 7 kb of sequence derived from ten stress response genes represented by nine ESTs. The same regions were also amplified and sequenced in the proposed diploid progenitors of white clover, *T. occidentale* and *T. pallescens*. Comparison of the amplicon sequences generated for each gene in this clone set allowed the identification of all three types of variation in the amplicons (that associated with homologs, homeologs and paralogs). Most interestingly, it was possible to use the proposed ancestor sequences to assign putative homeolog specific variants (HSVs) into ancestral sub-genomes (although this clearly worked better for *T. occidentale* than *T. pallescens*). This represents a robust way of both differentiating between allelic and non-allelic variants and also grouping them into consistent sub-genome classes (referred to as the O and P’ sub-genomes), but the process would be difficult to apply to many thousands of sequences.

Homeolocus organisation over tens/hundreds of kilobases has been examined in greater detail for a restricted number of loci in white clover. Hand et al. [12] sequenced eight bacterial artificial chromosome (BAC) clones representing four homeologous regions in genic areas of white clover (these were anchored by four of the aforementioned abiotic stress tolerance candidate genes). A total of 173 kb of overlapping sequence between the O and P’ sub-genomes was generated. Eighteen homeologous genes were identified in the overlapping sub-genome specific sequences and these exhibited conservation of order and orientation. A further eight genes apparently specific to one or the other sub-genomes were also observed, although this observation is complicated by the fact that homeolog copies of these genes may have been present in the alternative sub-genome just outside the windows of comparison. Levels of nucleotide identity in exonic regions of homeolog pairs ranged from 86% to 100%, with an average of 97%. In introns, nucleotide identity ranged between 66% and 100% with an average of 89%.

The constantly increasing throughput of next generation sequencing (NGS) technology is revolutionising our ability to describe both genome structure and variation. Recently, numerous draft whole genome sequences have been developed in higher plants, based either in part or entirely on NGS-based sequencing strategies. Plant species, many of which have large complex genomes with a history of ancient and recent duplications are still challenging to sequence. NGS is also rendering the transcriptome more accessible, with technologies such as Illumina-based RNA-Seq allowing sampling to depths capable of detecting relatively low-abundance transcripts in specific tissues and environmental conditions. This is a useful tool in both functional genomics and the annotation of genome sequences, but also can also provide a potentially stand-alone resource in the face of difficult genomes where whole genome sequencing is problematic. Another potential use of large scale transcript sequencing datasets is in high throughput, genome-wide SNP discovery. This was previously possible using Sanger-derived EST sequences, but the cost and comparatively low throughput nature of this strategy meant that, in comparison to NGS-based strategies, it was really only effective in relatively well-resourced model and crop plant species, where EST data could be aggregated from numerous sources.

The real potential of NGS for SNP discovery relies heavily on an ability to both discover and validate SNPs in an automated, high throughput manner. While the process of single nucleotide variant detection in allopolyploids is technically no more complex that the same process in homozygous diploids, the presence of multiple independently-segregating but potentially very similar sub-genomes requires extra layers of analysis in order to distinguish allelic and non-allelic variation. As illustrated by the above studies, this is certainly the case for allotetraploid white clover. While the approaches described in these studies [11,12] were both elegant and informative, the processes required to partition genetic variation into allelic and non-allelic types are unsuited to the validation of the thousands of SNPs required for downstream applications such as association mapping and genomic selection.

Recently, Trick et al. [13] described a strategy for NGS-based SNP detection in allotetraploid *Brassica napus* which allowed the discrimination of allelic single nucleotide variants (hemi-SNPs) and sub-genome-specific or homeolog-specific variants (inter-homeolog SNPs). The progenitor A and C sub-genomes of *B. napus* are very similar, with many genes expected to exist as homeolog pairs, the transcript sequences of which are expected to differ by only approximately 3.5% on average. As a result, such homeolog pairs will tend to co-assemble or cluster, even when relatively high similarity thresholds (~95%) are used. The strategy is based on the use of doubled haploid (DH) lines, in which the individual sub-genomes have been rendered homozygous. Allelic variants in a sub-genome will be fixed for one or the other alternative nucleotide. Nucleotide variants that distinguish homeolog pairs (inter-homeolog variants) originating from different subgenomes will still be present. When reads from a homeolocus in a single DH line are aligned relative to a reference sequence, nucleotide variants apparent between constituent reads of the alignment are either inter-homeolog variants or real allelic variants that differ between the subgenomes. In most alignment software, the consensus sequence generated from the alignments will represent such positions with ambiguity codes indicating the presence of more than one nucleotide. The key point in the strategy is the comparison of the consensus sequences for alignments from different DH genotypes.

Thus, any set of genotypes compared in this manner will always have the same consensus nucleotide interhomeolog variant positions. For positions where real allelic variation is the source of the ambiguity code in one genotype, comparison to a second genotype may reveal that the same position has a normal nucleotide code rather than an ambiguity code. This is likely to be the footprint of allelic variation at this position. Since, in essence, all that is required is the comparison of the consensus sequences resulting from at least two genotypes for each locus (defined by a reference sequence) this approach is amenable to being scaled up for numerous loci. Platforms such as Illumina can produce tens or hundred of millions of 100nt transcript reads per genotype, providing a source of genotype-specific sequences to align to a reference sequence, at a depth of coverage that allows the robust detection of nucleotide variants in the alignments.

The levels of nucleic acid identity for the subgenomes of white clover are similar to that of *B. napus*, so a similar strategy could be implemented with suitable homozygous genotypes. To our knowledge, no doubled haploid lines of white clover have been developed. However, inbred lines based on starting material in which the single-locus gametophytic self-incompatibility system has been overcome, presumably by the presence of one or more self-fertility (Sf) alleles, have been developed [14]. Originally, fourth and fifth generation inbred lines of four distinct origins (referred to as genotypes R, S, J and H) were developed by single seed descent. When these lines were intercrossed they yielded F_1_ hybrid progeny that consistently exhibited heterosis for a range of characteristics, supporting the generally inbred nature of the lines. Two of the lines were subsequently used in the first molecular marker based genetic linkage map of white clover [4], which was based on an F_2_ population of a cross between a fourth generation derivative of one line and a fifth generation derivative of another (population F2(I.4RxI.5 J)). Marker segregation data revealed a high retention of heterozygosity, with up to 60% of simple sequence repeat (SSR) markers exhibiting bands suggesting retention of heterozygosity in the parents.

In the study described here, we sought to develop publicly accessible EST and SNP resources for white clover. In the first instance we exploited the longer read capabilities (~400nt) of 454-pyrosequencing to generate an EST-like reference transcript set of approximately 70,000 sequences in a white clover inbred line. This was then used as a reference for two rounds of SNP discovery using shorter (76-101nt) Illumina reads, the first of which was based on generating approximately 14 million sequences from mixed cDNA samples of 24 white clover genotypes from a wide geographical range. In the second round of SNP discovery, we used the eighth generation derivatives of two of the sets of white clover inbred lines described above to mimic the genomic constitution that would be expected for doubled haploid lines. These lines have undergone three to four further generations of inbreeding than the lines described by Michaelson-Yeates et al. [14], and consequently, we started out from an assumption that the residual heterozygosity reported earlier [4] would be diminished.

Trick et al. [13] applied the MAQ aligner [15] and custom Perl scripts to identify and classify SNPs between DH lines at various read depths. In this paper we present a similar pipeline based on the combined use of BWA [16], Genome Analysis Toolkit [17] and SAMtools [18] both to identify single nucleotide variants, and tentatively classify their origin as allelic or homeolog specific.

## Results and discussion

### *De novo* sequence assembly of 454 reads

**Figure 1:**
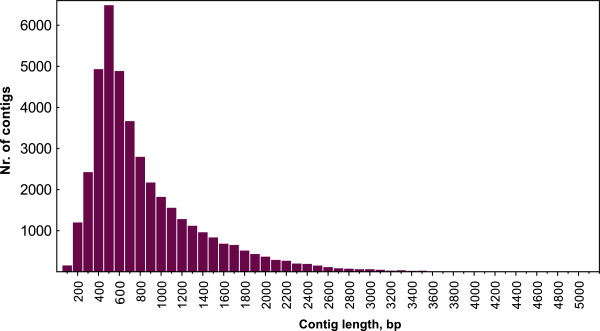
Length distribution of the 40,649 contigs of the reference transcript set.

To provide a reference transcript set for white clover, cDNA from mixed above-ground material of a single pot-grown plant of the inbred line S1 was subjected to sequencing on the Roche GS_FLX platform using Titanium sequencing chemistry, generating a total of 768,512 cleaned reads (mean length 300.21 nt). Assembly of these reads using MIRA [19] resulted in 43,902 contigs. These 43,902 contigs were built from 672,841 reads (87.55% of the original reads were incorporated into contigs). Of these, a total of 937 contigs incorporated more than 100 reads per contig, and 25 of these incorporated more than 1,000 reads per contig. A total of 13,726 contigs were built from two reads only. On average 13.29 reads were built into each kb contig length. The 43,902 contigs were clustered using the cdhit-est program of the CD-HIT package [20] by setting a similarity threshold of 0.95 and a word size of 8. The clustering resulted in a non-redundant set of 41,094 contigs. From these, 184 contigs shorter than 80 nt were removed. The remaining contigs were subjected to BLAST comparison against *Trifolium subterraneum* chloroplast sequence (GenBank Accession Nr: EU849487). Two hundred and sixty-one contigs gave hits with more than 90% similarity at an E-value threshold of >10E-50. These contigs were removed and the remaining 40,649 (mean length: 783 nt) were used for further analysis. The length distribution of the assembly following these processing steps is summarised in Figure 1.

Sequences of 454 reads that were not incorporated into contigs were recovered by using the information given in the *info_debrislist.txt* file provided by the MIRA assembler. The sequences were cleaned to remove vector and adapter contamination, and low quality and low complexity terminal sequencess (like polyA and polyT stretches) using the SeqClean script [21], resulting in a set of 62,152 cleaned 454 reads. Of these sequences, 7,897 were shorter than 80 nt and were removed. The remaining 54,255 sequences were clustered by CD-HIT at a similarity threshold level 0.95 resulting in 36,840 cluster-representative sequences. These sequences were subjected to a second clustering step along with the 40,649 contigs from the previous sequence assembly at a 0.95 similarity threshold level. The second clustering step retained 30,979 singlets. Eighty-three chloroplast specific sequences were identified by BLAST comparison with the *T. subterraneum* chloroplast sequence and removed. The remaining set of 30,896 non-redundant singlet sequences was used for further analysis. The mean length of the non-redundant singlet sequence set was 267.35 nt (min 81 nt, max 581 nt). Hereafter we refer to the combined set of contigs and non-redundant singlets (71,545 sequences) as the reference transcript set.

### Characterisation of the reference transcript sequences

In order to assess the overall quality of the 40,649 contigs and 30,896 non-redundant singlets, they were compared to the *Medicago truncatula* Gene Index (MtGI rel. 10.0) transcript database using the *tblastx* program of the BLAST package [22].

The top hits of the *tblastx* results were filtered into 3 categories using a Perl script:

(1) “Class I” hits: E-value < 1E-100, Score >200;

(2) “Class II” hits: E-value < 1E-20, Score >100;

(3) “Class III” hits: E-value >1E-20.

**Figure 2:**
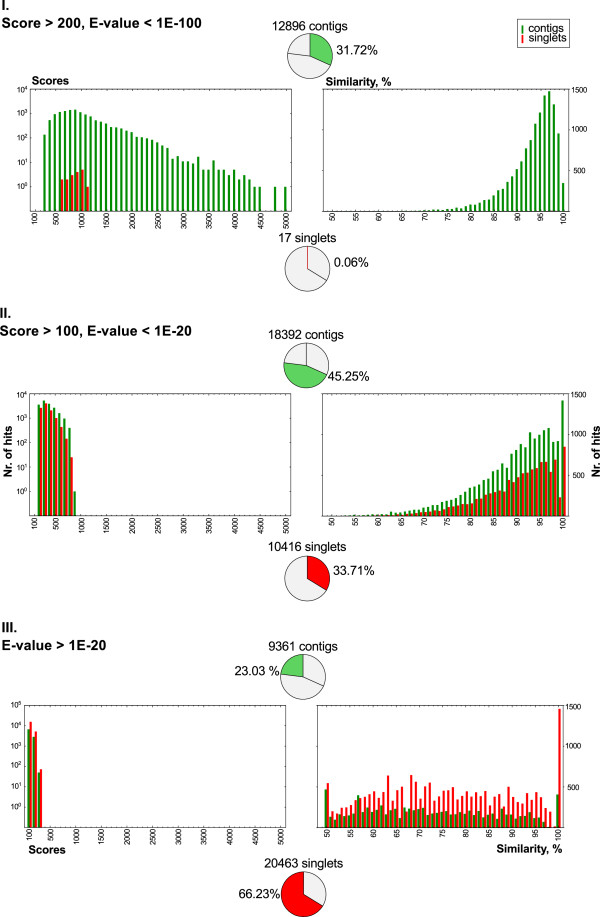
**Summary of BLAST analysis results.** All sequences of the white clover reference transcript set (40,649 contigs and 30,896 non-redundant singlets) were compared to a Medicago truncatula transcript sequence database (MtGI Rel. 10.0). The distribution of scores (left panel) and similarity (right panel) of the top-hits were plotted in the hit classes I, II and III (categorised according to the parameters given in the top left corner of each boxes). The number of query sequences belonging to the corresponding hit classes is given on the top (contigs) or under the similarity charts (singlets).

Seventy-seven percent of the contig sequences fell into the top two categories (12,896 in Class I and 18,392 in Class II) while the remaining 23% (9,361 sequences) were in Class III. For the singlet sequences the situation was reversed, with 66% of sequences (20,463) falling onto Class III, 33.7% into Class II and only 17 sequences (0.06%) in Class I (Figure 2). While this may be partially due to a difference in the gene content of the closely related model and forage legumes, it probably also indicates a generally lower level of sequence quality and reliability for the singlet sequences, and the appropriate caution was exercised in their use for the remainder of the study.

**Figure 3:**
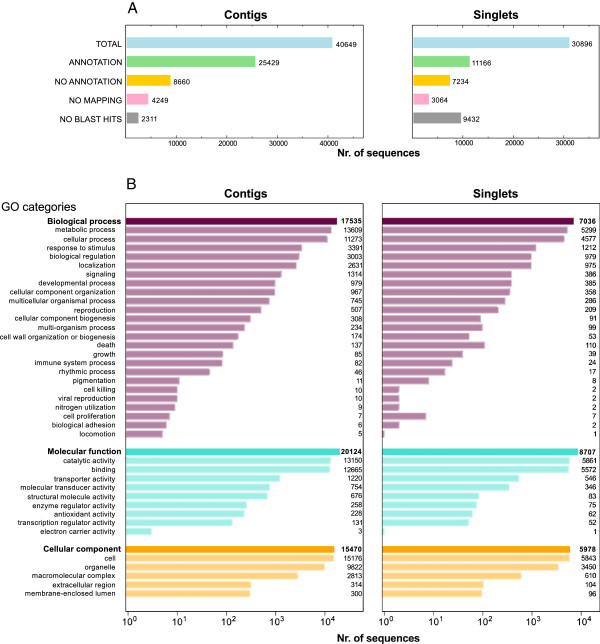
**(A,B). Annotation results. A**: Numbers and proportions of sequences successfully passing the subsequent steps of the annotation procedure of BLAST2GO as described in the Methods (contigs on the left and singlets on the right). No BLAST Hits: Sequences with no significant blastx hit to non-redundant protein database described in the methods. No Mapping: Sequences with blastx hits >1e-6, but for which no GO terms could be associated to the BLAST results. No Annotation: Sequences with blastx hits >1e-6 and associated GO terms lying below the GO annotation rule thresholds described in the Methods section. Annotation: Sequences with BLAST hits >1e-6 which were successfully assigned GO terms. **B**: Distribution of the main GO categories in annotated contigs (on the left) and singlets (on the right). Top level GO categories (Biological process, Molecular function and Cellular component) are shadowed. Bars in lighter colours represent sub-categories within the top level categories. The X-axis scale is logarithmic.

The sequences from the reference transcript set (40,649 contigs and 30,896 singlets) were subjected to functional annotation by comparison to a custom database of all available *Viridiplantae* protein sequences using the Blast2GO pipeline [23]. Using this approach 62.55% (25,429) of the contigs, but only 36.14% (11,166) of the singlets, could be annotated. Only 5.69% (2,311) of the contigs did not yield significant *blastx* results compared to 30.53% of the singlets. The total number of GO annotations was higher in the contigs than in the singlets, but the distribution of Gene Ontology categories (proportion of GO categories within the three main sub-ontologies (Biological process, Molecular function, Cellular component) were largely similar in contigs and singlets (Figure 3, A,B). A summary of the GO annotation is presented in Additional file 1, and a FASTA file of all 71,545 reference transcript sequences is presented in Additional file 2.

### SNP discovery in a mixed sample composed of highly divergent genotypes

In order to exploit the reference transcript set to identify SNP variation across the white clover genome we used two approaches. In the first instance, we generated approximately 14 million Illumina GAII reads from pooled cDNA of 24 very divergent heterozygous allotetraploid genotypes representing a pan-global geographic distribution of white clover, and thus likely to capture a significant proportion of the overall variation present in the cultivated genepool of the species. We aligned these sequences to the reference transcript set to identify SNP positions.

After cleaning, 13,213,625 Illumina reads (length 76 nt) were available for alignment to the reference transcript set. Alignment, mapping and variant calling were performed as described in the Methods section. Single nucleotide variants were identified by applying a read depth threshold of 6 and a base quality threshold of 20. The process resulted in the identification of 89,503 SNPs, (77,593 in the contigs, 11,460 in the singlets). Of the 89,503 SNP variants 77,305 were due to ambiguity codes in the read consensus of aligned Illumina reads, the remainder (11,748) were departures from the reference. In all, SNPs were detected in 20,159 reference sequences (15,451 contigs and 4,708 singlets). The average read depth at the SNP positions was 25.69 (median value 16) in the case of the contigs and 27.35 (median value 15) in the case of the singlets. Hereafter we refer the mapping assembly representing this dataset as the DL assembly.

### SNP discovery and classification using white clover inbred lines

In the second instance, we generated approximately 50 million reads for each of two further white clover inbred lines on the Illumina HiSeq 2000 platform and aligned these to the reference transcript set.

For identification of SNPs between the two white clover inbred lines, cDNA from individual plants was used. One of the lines, SC was nearly identical to the S1 line used for the reference transcript assembly, as it was sourced from the same batch of seeds produced by a single self-fertilisation event. Line J5 was derived from a different, genetically distinct background, with the expectation of reasonable levels of nucleotide polymorphism relative to SC confirmed by some preliminary analysis using two AFLP assays (results not shown).

Illumina reads (101 nt long, 48,061,093 from SC and 50,378,626 from J5) were aligned to the reference transcript sequences using the pipeline described more fully in the Methods section. From a merged alignment that contained Illumina reads from both genotypes (hereafter referred as IL assembly) we separately called SNPs for each read group in positions where there was read depth of at least 6, applying a base quality threshold of 20. This produced two lists of variants (based on the SAMtools pileup files), one for each genotype. Conceptually, these variant lists contain positions where variants (ambiguity consensus codes or departure from the reference base) occur in both genotypes and positions where variants are observed in one genotype but not the other. We compared the two variant lists, and for the latter class we examined the merged alignment in regard to the opposing, non-variant genotype, and accepted the non-variant status where there was a read depth of at least 6 in the non-variant read group. This produced a “high confidence” set of variant calls over the two genotypes that were supported by a minimum read depth of 6 in each genotype. This variant set comprised instances in which variants were observed in either both genotypes, or exclusively in one genotype or the other.

In addition, for completeness, we also recorded all instances in which there was a variant in one genotype and not the other, where the second genotype had a read depth of lower than 6, including those for which there was no read coverage (ie. read depth of 0 to 5). Finally, we recorded instances in there was no apparent variation in either SC or J5, but where there was an apparent departure from the reference sequence.

This analysis identified 172,531 independent SNPs in alignments to 28,210 sequences from the reference transcript set sequences (150,597 SNPs in 20,674 contig sequences and 21,934 SNPs in 7,536 singlets). No variants satisfying our filtering parameters were found in the remaining reference transcript set of 19,975 contig sequences and 23,360 singlet sequences. The positions and nucleotide composition of the variants identified in both the DL and IL assemblies are listed in Additional file 3. Variant positions are also embedded into the headers of the appropriate sequences in the FASTA file presented in Additional file 2.

**Figure 4:**
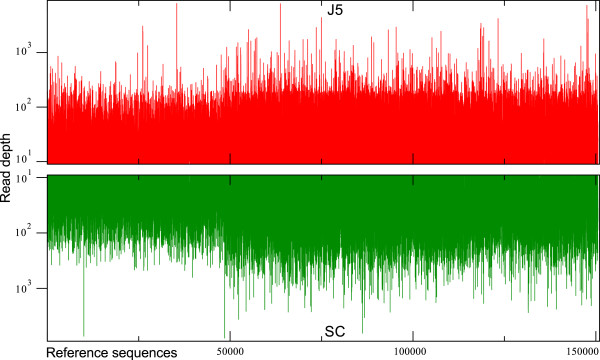
**Read depth of the J5 and SC read groups in the merged mapping assembly.** Plots of the actual read depths at 150,597 variant positions on the reference transcript contigs in genotypes J5 (top, red) and SC (bottom, green). (Note that read depth values were capped by the assembly software at 8,000).

In the case of the reference contigs, the mean read depth at the SNP positions was 47.99 (median value 27) for the J5 read type and 53.79 (median value 28) for the SC read type (Figure 4). Perhaps surprisingly, given our assumption that they might have an inherently lower quality, similar short read coverage could be detected for the reference singlets, where the average read depth was 51.35 for the J5 read type (median value 19) and 49.27 for the SC read type (median value 19).

**Figure 5:**
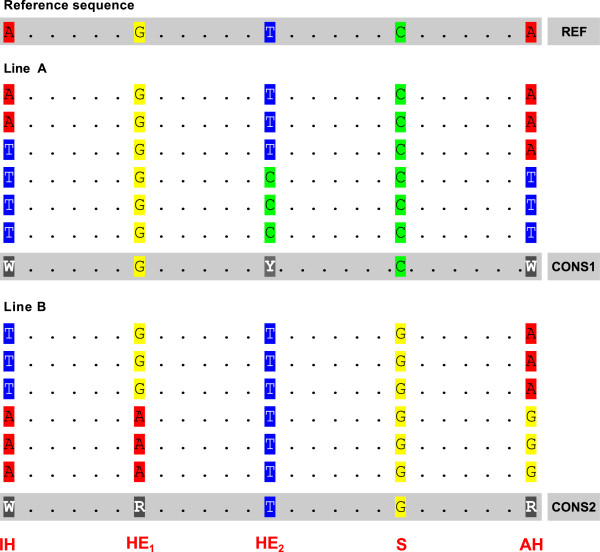
**Model of possible SNP types in an allotetraploid species.** Possible configurations of single nucleotide variants when sequences from two homozygous lines (Line A, Line B) are compared to a reference sequence. IH: Inter-homeolog SNP; HE1: Hemi-SNP, variant in Line B; HE2: Hemi-SNP, variant in Line A; S: Simple SNP; AH: Asymmetric Hemi-SNP. CONS1 and CONS2 indicate the consensus sequences from Line A and Line B respectively, REF indicates the reference sequence*.*

Subsequent to variant position identification, we employed the set of principles outlined by Trick *et al.*[13] to tentatively partition SNPs into classes attributable in origin to homeolog-specific or allelic variation. Using custom Perl scripts, the nucleotide consensus of both read type at each variant position from the variant lists for the two inbred lines was compared regarding ambiguity type and relation to the reference base, and variants were assigned to different SNP categories (summarised in Figure 5) as follows:

(1) Inter-homeolog SNPs (IH)

representative of homeolog specific variation. These comprise variants with the same ambiguity consensus sequence in both the J5 and SC read types (e.g. T/W/W, where the first character is for the reference base and the second and the third characters are for the SC and J5 read types respectively).

(2) Hemi SNPs (HE)

representative of allelic variation. In these cases, no variant can be detected in one of the read types (i.e. the consensus sequence is non-ambiguous in one of the read types), but the opposing read type exhibits variation (ambiguous consensus) at a given position (e.g. T/T/W). A subcategory of this class occurs for variants where the two read types have different ambiguous consensus sequences (typically, the two read types share one common allele, e.g. T/W/Y). We categorized these as **Asymmetric hemi-SNPs (AH).**

(3) Simple SNPs (S)

This class of variants represents inter-genotype polymorphism where the two read types display different unambiguous consensus nucleotides (e.g. T/T/A). This class could arise from several different sources, eg., effective diploid status at that locus or co-positional allelic variation in both homeolog genomes.

(4) Irregular SNPs (X)

In addition to the three main SNP classes, further minor variant classes were identified that could not easily be assigned to the above classes, but may represent real polymorphism, and thus we recorded these in a single category. This included: (i) Instances in which an ambiguity code was observed in one read type, but low (0 to 5 ) read depth occurred in the opposing read type; (iI) A class of variant position where both read types have the same unambiguous consensus sequence which differs from the reference sequence - in most cases the reference base was ambiguous at such position e.g. M/C/C; (iii) Positions where one or both read types exhibit consensus of more than two constituent bases (e.g. W/W/B or W/B/B).

From the total of 172,531 variants identified, 73,571 (42.64%) were categorised as Hemi-SNPs, representative of allelic variation. A further 53,663 (31.10%) were categorised as Inter-homeolog SNPs and 19,953 (11.56%) were classified as Simple SNPs. The remaining 25,344 variants (14.69%) fell into the Irregular SNP category. Nearly three quarters of the latter (18,058) were of the variety in which there was an ambiguity code representing a variant in one genotype, but low or no coverage in the other, making it impossible to assign to a definitive category using our threshold criteria. The majority of the remainder involved positions where J5 and SC exhibited the same nucleotide but differed from the reference base. These results are summarised in Table 1. Detailed information regarding position on the reference sequence, SNP type and read type specific consensus nucleotide for each SNP is given in Additional file 3.

**Table 1:** SNP categories found in the IL dataset

**SNP types**	**Contigs**		**Singlets**	
***Sub-categories***	**Nr.**	***%***	**Nr.**	***%***
**Hemi-SNPs**	**65147**	*43.26*	**8424**	*38.41*
**Inter-homeolog SNPs**	**46780**	*31.06*	**6883**	*31.38*
*Congruent consensus*	*46765*		*6881*	
*Non-congruent consensus*	*15*		*2*	
**Simple SNPs**	**18534**	*12.31*	**1419**	*6.47*
*CONS1 is the same as REF*	*18007*		*1314*	
*CONS2 is the same as REF*	*525*		*105*	
*Others*	*2*		*0*	
**Irregular SNPs**	**20136**	*13.37*	**5208**	*23.74*
*Low coverage (0 to 5) in the opposing read type*	13583		4461	
*Departure from REF*	*6539*		*747*	
*More than 2 alleles in one genotype*	14		0	
**Total**	**150597**		**21934**	

One interesting feature of this study is that the reference sequence was generated from the same inbred lineage as one of the test genotypes (SC). As such, there are additional expectations in terms of the types of SNP configurations that should be observable in the partitioning analysis relative to a situation where the reference data were aggregated from numerous different sources. The most obvious expectation relates to the simple SNPs, in that the test genotype SC should share the same nucleotide variant as the reference sequence in this situation (eg G/G/A). In fact, this expectation was met in the vast majority (96.8%) of the 19,953 simple SNP calls (Table 1).

A second, slightly more complex set of expectations relates to the Hemi-SNP calls. Part of the complexity in this case comes from the fact that we have allowed ambiguity codes to be used in the reference transcript assembly. Because of this, variant positions will sometimes be represented as ambiguity codes in the reference contigs, but this will not always be the case, due to the lower sequence depth of the 454 sequence dataset, and in these cases, one variant or the other will be represented. This is not an issue for the singleton sequences, where such ambiguity codes cannot occur. Theoretically, ambiguity codes appearing in the reference contigs should be matched ambiguity codes in SC at that position, since the two genotypes are near-identical. A more common occurrence is where an ambiguity code is observed in SC but not in the reference sequence. In this case the nucleotide from the reference sequence should represent one of the two constituent bases of the ambiguity code. When these factors are taken into account, of the 73,571 Hemi-SNPs identified, 70,353 (95.6%) fell into four non-problematic subcategories (bold-faced in Table 2) that accorded to expectations of identity between the reference sequence and SC, while the remaining 3,218 (4.4%) fell into categories that were in some way problematic, due to either apparent discord between the reference nucleotide and that in SC, the occurrence of three nucleotide variants rather than two, or some combination thereof. The latter categories may represent a mixture of sequencing, alignment and variant calling error, deviations from homozygosity, triallelism and paralog incorporation.

**Table 2:** Sub-categories of Hemi-SNPs in the IL dataset

**Code**	Examples*		**Contigs**	**Singlets**	**Total**
	**Consensus (REF/SC/J5)**	**Underlying bases**			
**H-A1**	**G/G/R**	**g | g | g**	**35756**	**4110**	**39866**
		** | | a**			
H-A2	G/A/R	g | a | a	1145	245	1390
		| | g			
H-A3	K/G/K	g | g | g	930	0	930
		t | | t			
H-A4	K/G/R	g | g | g	28	0	28
		t | | a			
H-A5	G/T/R	g | t | g	1	10	11
		| | a			
H-A6	Y/C/K	c | | g	10	0	10
		t | | t			
H-A7	C/T/K	c | | t	73	11	84
		| | g			
**H-B1**	**G/R/G**	**g | g | g**	**14348**	**1801**	**16149**
		** | a |**			
**H-B2**	**Y/Y/C**	**c | c | c**	**2869**	**0**	**2869**
		** t | t |**			
**H-B3**	**G/K/T**	**g | g | t**	**9320**	**2149**	**11469**
		** | t |**			
H-B4	R/R/C	a | a | c	5	0	5
		g | g |			
H-B5	G/R/T	g | g | t	3	0	3
		| a |			
H-B6	M/S/C	c | c | c	19	0	19
		a | g |			
H-B7	R/S/C	g | g | c	4	0	4
		a | c |			
AH-1	S/S/R	g | g | g	91	0	91
		c | c | a			
AH-2	K/W/K	t | | t	4	0	4
		g | a | g			
AH-3	C/S/M	c | | c	279	43	322
		| g | a			
AH-4	C/M/R	c | a | a	245	50	295
		| c | g			
AH-5	C/R/S	c | g | g	17	5	22
		| a | c			
Total			65147	8424	73571

*The column contains one example from all of the possible similar configurations.

Bold setting indicates non-problematic subcategories as described in the main text.

Code H-Ax indicates no variant nucleotides in SC, variant in J5;

Code H-Bx indicates variant nucleotides in SC, no variant in J5;

Code AH-x indicates asymmetric variation in both SC and J5.

Similarly, for the IH SNPs, there is an expectation of congruence between all three genotypes (the reference and both inbreds). Deviations from this expectation occurred in only 17 (0.03%) of 53,663 IH SNP calls.

While, given our model assumptions, the origin of Hemi-and IH SNPs is relatively clear in terms of allelic vs. homeolog specific variation, the origin of Simple SNPs is perhaps less clear. Instances in which Simple SNPs are arising from loci for which no clear homeolog copy exists (effectively diploid loci), or from the transcriptional suppression of one homeolog copy in favour of another are likely to be represented in our dataset by reference sequences in which only Simple SNPs between the inbred lines are present. From 8577 reference transcript sequences containing simple SNPs, 2136 contained only Simple SNPs (about half of these contain only one SNP in the entire contig), meaning that up to one quarter of contigs containing simple SNPs could come from the aforementioned sources. In the majority of occasions, Simple SNPs occur in contigs also containing Hemi- and IH-SNPs, indicating that both homeologs are present and expressed in the genotypes studied. Hand et al. [11] found that approx 22% of SNP positions that they surveyed for ten independent genes between a number of mapping population parents contained polymorphisms in both sub-genomes, generally exhibiting the same nucleotide variants (eg AT[O]AT[P’] using their nomenclature, where O and P’ indicate the respective subgenomes). For many of the positions appearing as Simple SNPs in our data, it’s likely that, in the process of homogenisation associated with inbreeding, opposing allelic variants were fixed in the inbred lines (eg. AT[O]AT[P’] homogenised to TT[O]TT[P’] in one inbred line and to AA[O]AA[P’] in another. Thus, it is reasonable to hypothesise that the majority of Simple SNPs are representative of a type of allelic variation.

### Homozygosity in the J5 and SC test genotypes

One of the major assumptions underlying our attempt to partition SNPs into allelic and non-allelic variants is that the two genotypes under comparison are completely homozygous. In this study, we have used eighth generation inbred lines derived from self-fertile white clover genotypes J and S [14] in which the genetically determined self-incompatibility system has broken down. Theoretically, eighth generation inbred lines, should retain less that 1% residual heterozygosity. However, it has been observed that inbred lines generated from obligate outbreeders in which a genetically determined self-incompatibility system has been circumvented tend to exhibit levels of heterozygosity in excess of that predicted at any particular generation of selfing. This tendency has been specifically demonstrated for the white clover lineages involved in this study. As previously outlined, the first genetic linkage map of white clover was developed using a fifth generation inbred line derived originally from self fertile genotype J, and a fourth generation inbred line generated from self fertile genotype R. Segregation of co-dominant SSR markers in the F_2_ mapping population suggested residual heterozygosity levels as high as 60% [4]. Thus, while three to four further generations of inbreeding are likely to have increased the extent of homozygosity, a residual heterozygosity level of <1% for J5 and SC is unlikely. Observation of the segregation patterns of SNPs identified in this study in a bi-parental mapping population derived from the two test genotypes would have been a useful method for determining the extent of residual heterozygosity. Unfortunately, we did not have such a population. As an alternative method of assessing the probable extent of heterozygosity, we manually examined alignments of Illumina reads from the IL (merged J5/SC) mapping assembly using 400 randomly chosen sequences from the reference transcript set (corresponding to ~2% of the 20,675 in which SNPs were detected). Our hypothesis was that homozygous loci would consistently exhibit no more than two detectable haplotypes per genotype in the alignments, corresponding to the ancestral sub-genomes, while loci exhibiting retention of heterozygosity would tend to exhibit three to four haplotypes due to combined allelic and inter-genomic variation. We developed a rigorous set of rules for this visual examination that took into account the fact that the alignments consisted of sequences of only 101 nt, limiting the ability to identify “in-phase” variants to instances in which these occur on the same constituent read of an alignment. In the majority of cases, a nearly equal distribution of two haplotypes could be detected, of which the SC-derived haplotype was similar to that of the reference sequence (as expected). After inspecting the mapping assemblies of the 400 contigs, more than two haplotypes were detected in only 25 (6.25%) of the examined contigs, with three or more haplotypes occurring in both genotypes on 8 occasions, in J5 alone on 14 occasions, and in SC alone on 3 occasions. Although we hesitate to use this approach to assign a specific estimate for the level of retained heterozygosity on a genome wide level, it supports the hypothesis of an allotetraploid state, consisting largely of two extensively homozygous homeologues for both (J5, SC) test genotypes.

In addition to demonstrating that residual heterozygosity is limited in the inbred lines, this analysis suggests that, in general, the expectation that homeolog copies will tend co-assemble in the reference transcript assembly and subsequent mapping assemblies is valid. Available data suggest that the average nucleotide identity levels in white clover are as high as 97% [12]. With iterative clustering steps at a 95% similarity level during the *de novo* assembly phase, our process should generally achieve co-assembly of homeolog copies, although nucleotide similarity levels as low as 86% were previously reported [12], and such homeolog pairs may not co-assemble at this similarity level. Less stringent clustering parameters would remediate this effect, but increase the frequency of the incorporation of paralog copies. While not an in depth examination of paralog co-assembly, the examination of the 400 contigs suggests co-assembly of multiple paralogous copies was also not widespread, since this would also yield more than two apparent haplotypes.

### Comparison of variants from divergent and inbred lines

**Figure 6:**
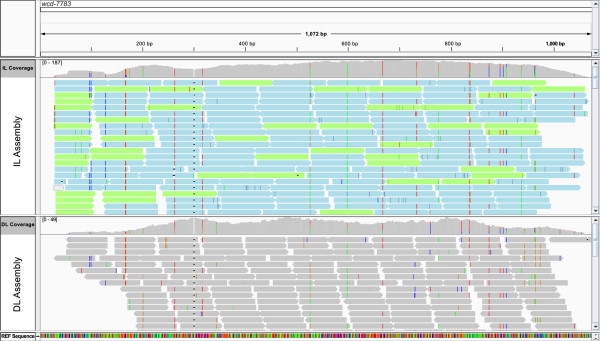
**Mapping assemblies and variant positions in the IGV graphical alignment viewer window.** The reference transcript contig wcd-7783 is presented as an example. The two main panels in the middle show the IL and the DL mapping assemblies with read depth plots on the top. Illumina reads from the J5 and SC genotypes are shown as bars in blue and green respectively, reads from the DL assembly are grey. Vertical bars in different colours represent variant positions with different base compositions. The reference sequence (REF) is also represented on the bottom panel as vertical coloured bars.

**Figure 7:**
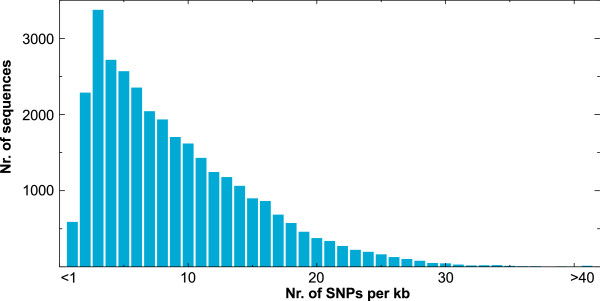
**SNP density distribution.** The density of variant positions on each SNP-containing reference transcript sequence (23,056 contigs and 8,659 singlets) was calculated as number of single nucleotide variants per sequence length in kb.

In order to identify the total number of independent SNP positions identified in this study, we made a direct comparison between the variant lists arising from the divergent (DL) and inbred (IL) line mapping assemblies. Comparing the 172,531 SNPs from the inbred lines to the 89,503 SNPs from the divergent lines revealed that 52,730 SNPs were positionally common between the two datasets, with 119,801 variants occurring only in the inbred line dataset and 36,323 variants specific to the divergent lines (an example with common and read-group specific variants in a graphical alignment viewer window is given in Figure 6). Thus, in the course of this study a total of 208,854 independent variant positions were identified in 31,715 reference transcript sequences (23,056 contigs, 8,659 singlets) comprising a total sequence length of 25,690.57 kb (Additional file 3). This corresponds to an average density of 8.46 SNPs per kb, or one SNP per 118.25 bp of sequence. The density distribution of SNPs on the reference sequences is shown in Figure 7.

Examining the DL assembly at the variant positions that were exclusively detected in the IL assembly, we concluded that the main factors responsible for a lack of corresponding variants were either total absence of short read coverage or an insufficient read depth in the DL assembly. However, in 10,485 cases, positions that were found to be variant in the IL assembly exhibited no variation in the DL assembly at read depth levels of greater than 6. SNPs were exclusively detected in the DL dataset at 36,323 positions. Of these, 16,225 were located in non-variant positions in the IL dataset supported by a read depth of greater than 6, and the remaining 20,098 positions exhibited low read depth and/or low base quality values that prevented the calling of variants in the IL assembly.

The co-incidence of a large number of variants between the DL and IL assemblies offered the opportunity to compare the frequency of occurrence of the major SNP categories from the IL analysis in the DL dataset. Variant positions categorised in the IL assembly as IH SNPs are more representative of ancestral variation and thus should have representation approaching 100% in comparable positions in the DL assembly. Conversely, Hemi-SNPs (and Simple SNPs) are representative of allelic variation, and thus one might expect that there are allelic variants represented in the IL assembly that are NOT present in the variation captured in the DL assembly. In order to test this, we identified all variant positions in the IL assembly supported by read depths of 6, 8 and 10 in each individual inbred line, for which there was matching read depth support in the DL assembly. We then identified whether variants were also present at these positions within the DL assembly. At the highest stringency level, which required read depth of at least ten in each inbred line and the DL assembly, there were 22,220 IH SNPs from the IL assembly for which there was sequence coverage in the DL mapping assembly. Of these, 19,626 positions (88.33%) were also variant in the DL assembly (Table 3). By comparison, of the 26,900 Hemi-SNP positions from the IL assembly at this read depth level, 18,793 (69.86%) were also variant in the DL assembly. Thus, at these very well supported positions, ~90% of IH SNPs were represented in the DL assembly as opposed to ~70% of Hemi-SNPs. At lower read depths (6 and 8), representation of individual classes was slightly lower, but the difference of ~20% between the categories was maintained. Simple SNPs were consistently represented at lower levels still (62.37% at a read depth of 10). These significant differences in the representation of categories derived from allelic and homeolog specific variation are in accordance with the expectations described above, and also offer support for the ability of the analysis in the inbred lines to successfully partition these two types of variation.

**Table 3:** Comparison of SNPs in the IL and DL datasets at different read depth levels

**Read depth**	**IH**		**HE**		**S**	
Nr. of common positions*						
**6**	34280		43743		10469	
**8**	27514		34163		8025	
**10**	22220		26900		6277	
SNP variants in the DL assembly**						
		*%*		*%*		*%*
**6**	28756	*83.89*	28393	*64.91*	5992	*57.24*
**8**	23537	*85.55*	22759	*66.19*	4767	*59.4*
**10**	19626	*88.33*	18793	*69.86*	3915	*62.37*

IH: Inter-homeolog SNPs, HE: Hemi-SNPs, S: Simple SNPs.

*Number of reference sequence positions where short read coverages were at or above the applied read depth threshold (given in the first column) for both read types in the IL assembly, as well as in the DL assembly.

**Number and percentage (in italics) of DL variants with ambiguity consensus code in matching positions. Note that no base quality filtering was applied for this comparison when searching for matching DL variants.

One potential problem in using transcript data for SNP discovery in allopolyploids is the possibility of transcriptional suppression of genes from different sub-genomes in different lines. On an individual variant basis this could result in the mis-identification of interhomeolog variation as allelic variation. Interestingly, the pipeline described in this study can address this problem at the discovery stage, since the identification of IH SNPs in alignments to any reference sequence indicates the expression of both homeolog copies of that sequence in both inbred lines. In addition, the identification of HE SNPs from both inbred lines for a particular reference sequence also indicates the expression of both homeologs. In this study, of the 28,210 sequences originally identified as containing variants using the IL assembly, 74% (20,994) showed clear expression of both (O and P’ sub-genome-derived) homeolog copies in both of the inbred lines using these criteria. Amongst the remaining 26% of reference sequences, many variants falling below the read depth criteria (Irregular category SNPs) were identified, indicating probable expression of both homeologs. Excluding sequences containing Irregular category variants for this reason, only 11% of reference sequences (3163 of 28210) clearly exhibit the footprint of possible homeolog-specific transcriptional suppression in one inbred line. In these cases, no IH SNPs were identified and all HE and S variants were derived from one line, indicating possible suppression in the other. This suggests that homeolog-specific transcript suppression is not a prevalent feature of this dataset, and that the analysis pipeline employed can identify potential cases, reducing the need for subsequent validation to account for this phenomenon.

### Potential for haplotype reconstruction and progenitor comparison

As previously outlined, Hand et al. [12] identified and sequenced four pairs of partially overlapping BACs representing the O and P’ subgenomes of white clover. In a total overlap of 173 kb, eighteen homeologous pairs of genes were identified. At the time of writing, these represented the best set of matched homeolog gene sequences available from white clover, and we decided to compare our results to them, with the specific objective of assessing the potential for our pipeline to successfully partition sequences into their respective sub-genomes. We performed BLAST-based comparisons of the published sequences of these eight BAC clones to the contigs from the reference transcript set, and identified nine sequences from the latter which gave good full-length hits to predicted coding sequences on the BACs, allowing them to be identified as unequivocal homologs/homeologs. We then examined the reference transcript sequences for SNPs identified using the IL mapping assembly. For three of these sequences, no SNPs had been identified. For one sequence we found only two SNPs, in the Irregular category, indicating that they were supported by insufficient coverage. The remaining five transcript sequences contained SNPs that had been identified at sufficient coverage to be categorised as Hemi-, IH- or Simple-SNPs. One of these contained only 4 Simple SNPs. The remaining four transcript sequences contained IH- and Hemi-SNPs as well as the other categories (see details in Additional file 4).

These alignments present the first opportunity to examine the sub-genomic origin of IH SNPs identified by our analysis. Of the 34 IH SNPs in the 4 transcript sequences, 17 had equivalent sequence coverage in the BAC-derived gene sequences. Nucleotide variation between the BAC-derived sequences from alternate sub-genomes matched IH variation in the inbreds on 12 occasions. At the remaining 5 comparable IH SNP locations, no variation was observed between BAC-derived sequences. This figure is not unreasonable given that Hand et al. [11,12] found that there was a high degree of coincidence between allelic SNP and homeolog specific variation, and a general concordance of nucleotide variants at sites where this was occurring. They quantified the proportions of co-locating allelic SNPs and HSVs, and found that, in their dataset, on average, 28% of HSV locations experienced simultaneous allelic SNP variation. This rose to 75% in one individual case. The effect of this is that, rather than there being a clearly identifiable set of homeolog specific variants that reflect the progenitor state of each sub-genome, homeolog specific variation in white clover is much more variable. This conclusion has specific ramifications for our attempts to partition the observed SNP variation in our inbred lines into allelic and homeolog specific variants, and the general utility of this information. The most salient feature from a utility point of view is that our analysis should not be viewed as a comprehensive catalogue of homeolog specific variation for the sequences surveyed. The process we implement will specifically capture instances of interhomeolog variation between these two lines.

**Figure 8:**
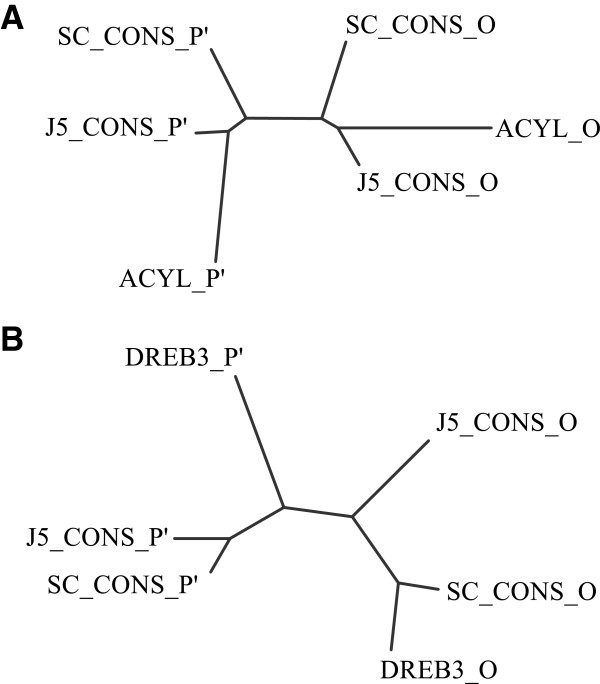
**(A, B) Unrooted phenetic trees from sequence alignments of haplotype-specific consensus sequences and sub-genomic sequences. A**: Acyl-coA oxidase: ACYL_O and ACYL_P’ indicate the published sequences for the O and P’ sub-genome derived sequences GU443966_9 and GU443966_17 respectively. Reconstructed haplotypes representing consensus sequences from the genotypes SC and J5 are named as SC_CONS_O, J5_CONS_O (haplotype O) and SC_CONS_P’, J5_CONS_P’ (haplotype P’) respectively. **B**: Dehydration responsive element binding protein “DREB3”: DREB3_O and DREB3_P’ indicate the published sequences for the O and P’ sub-genome derived sequences GU443966_13 and GU443966_21 respectively. Reconstructed haplotypes representing consensus sequences from the genotypes SC and J5 are named as SC_CONS_O, J5_CONS_O (haplotype O) and SC_CONS_P’, J5_CONS_P’ (haplotype P’) respectively.

Given the limitations inherent in exploring the sequence variability at isolated nucleotide positions, we decided to extend the analysis to encompass multiple SNP positions simultaneously by attempting to reconstruct the underlying haplotypes for the inbred lines across two of the gene sequences. We chose the transcript sequences that, given the SNP density and distribution (generally one SNP per <100 bp, evenly spaced throughout the sequence), and the read coverage in J5 and SC, it was most likely that we would be able to manually reconstruct the haplotypes for the full length of the underlying sequences. Reference transcript sequences wcd-12699 and wcd-2781 both possess 16 SNPs in the IL assembly (Additional file 4). For both sequences this allowed the reconstruction of two uninterrupted, contiguous haplotypes from each inbred line, presumably corresponding to the two sub-genomes, spanning just over 900 bp in wcd-12699 and 2.3 kb in wcd-2781. These were subsequently aligned to the BAC-derived O and P’ genome specific coding sequences of genes encoding dehydration responsive element binding protein (*DREB3*) and acyl-coA oxidase respectively (alignments are shown in Additional file 5). Interestingly, for the wcd-2781/acyl coA oxidase comparison, the initial alignment shows that, in addition to SNP variation, a large InDel event spanning ~180 bp exists between the O and P’ sub-genome copies, and this is present in the haplotypes reconstructed from the inbred lines, allowing immediate identification of the sub-genomic origin of the four reconstructed haplotype sequences. For both this and the wcd-12699/*DREB3* comparison, sequences were edited to remove non-overlapping regions and realigned to generate simple unrooted phenetic trees to test whether the reconstructed haplotypes would cluster with the different sub-genome specific sequences from the BACs. In both cases the resulting trees exhibited two distinct clusters (Figure 8), each with one J5 and one SC haplotype clustering with one of the BAC-derived sequences representing the O and P’ subgenomes. Although limited to two sequences, this demonstrates the potential for both haplotype reconstruction and progenitor-based comparison to further elucidate homeolog relationships based on the type of data generated during this study. The read lengths of NGS technologies are generally increasing, and even at the time of writing, increased single end read lengths, and the ability for paired end sequencing could make haplotype reconstruction more feasible, especially given the reduction in complexity offered by inbreeding-derived homogeneity.

Hand *et al.*[11] pointed to the need to identify a core set of SNPs in white clover that are specific to individual sub-genomes and do not coincide with homeolog specific variants. They suggest a strategy of “extensive sequence-based sampling of germplasm collections using highly parallel technology” combined with the elegant progenitor-based comparison method they describe, largely driven by *T. occidentale*, since *T. pallescens* seems to be less similar to the P’ sub-genome The results above suggest a complementary strategy. Were it possible to assemble a reasonable-sized collection of divergent inbred lines (exhibiting sufficient homozygosity), they could be subjected to the pipeline described in this study. Inter-homeolog SNP calls would in this case be based on only those ambiguity codes occurring in all of the genotypes under examination, eliminating homeolog specific variant sites at which allelic variation was also occurring, thus producing a core set of ubiquitously present homeolog specific variants. The success of the strategy would largely be determined by the number of lines examined, but, even using advanced generation inbred lines derived only from the four genotypes described originally [14], it is probable that a significant number of sites exhibiting allelic variation could be discounted. Obviously, such a process cannot partition the variants into specific sub-genomes, but combining it with the progenitor comparison approach and more automated methods of haplotype reconstruction from mapping assemblies would significantly extend the utility of the approach in this respect.

## Conclusions

The main goal of this study was to develop a significant publicly available SNP resource for white clover. We have developed, and partially annotated a reference transcript set of over 70,000 sequences for white clover, and used these to identify over 200,000 independent SNPs in approximately 45% of the reference transcript sequences. In recognition that the utility of these SNPs is partially based on whether they are truly allelic or sub-genomic in origin, we have attempted to partition a significant portion of them into categories related to these origins, and examined the most significant parameters likely to effect the accuracy of these categorisations.

The SNP data processing and analysis pipeline presented in this study can easily be adapted to SNP discovery projects in other allopolyploid species. Based on the principles outlined by Trick *et al.*[13] it differs in that the Perl-script enhanced comparison of genotype specific variant lists is obtained and further processed by different utilities of the SAMtools package rather than the somewhat older MAQ, overcoming several problems related to implementing this type of analysis with the latter. SAMtools takes alignments in the generic SAM (Sequence Alignment/Map) format, which is natively supported by various short read aligners. Unlike MAQ, SAMtools is under active development, is more likely to be able to handle newer file formats from different sequencing platforms, and produces output formats that are adopted by a wide range of downstream application software.

An additional advantage of using SAMtools is that short reads from different genotypes can easily be mapped and aligned onto the same set of reference sequences, while variant calling can be performed both globally as well as specifically for any particular genotype, or for a set of genotypes from combined alignments. This can be effectively utilised in the separation of allelic and sub-genomic variants in two or more genotypes, and we have demonstrated how this type of analysis could be extended to identify a core set of homeolog specific SNP variants that would be useful for distinguishing the sub-genomic origin of white clover transcripts, while simultaneously providing an even more comprehensive collection of potential allelic variants for genome-based analysis in white clover.

## Methods

### Plant materials, RNA extraction and sequencing

Three genotypes from two of the sets inbred lines originally described by Michaelson-Yeates et al. [14] were used for this study. All were eighth generation derivatives of lines S and J. Line S1 was used for the generation of the reference transcript set, while lines SC (a full sib of S1) and J5 were used to generate shorter reads for SNP discovery (via alignment to the S1 reference reads). For S1, RNA was extracted from a mixed tissue sample (including leaves, stems, stolons and flowers) of above ground parts of a plant grown pot that was placed in darkness for 24 hrs prior to material collection. For lines SC and J5, RNA was extracted from leaf material of a pot grown plant with similar darkness treatment. A second set of material comprised a bulk RNA sample isolated from leaves of individual plants derived from 24 highly divergent white clover varieties covering a broad geographical range. Plants were derived from the following varieties: Alice, Aran, Aberdai, Crusader, Goliath, Reisling, Apis, Dacia, Nanouk, Konitsa (Europe), Grasslands Huia, Grasslands Bounty, Grasslands Kopu II, Grasslands Prestige, Will, Grasslands Tribute, Grasslands NuSiral, Haifa (Australasia), Mineooha, North White (Japan), Zapican, Crescendo, Durana, Oklahoma (Continental America). Total RNA was extracted from all materials using the Qiagen Plant RNA Extraction Kit, according to manufacturers instructions. All cDNA synthesis and sequencing was performed by the company GATC Biotech AG (Constance, Germany). The SMART cDNA synthesis kit (Clontech) was used for cDNA library construction. For the reference transcript assembly, sequencing was performed on a single picotitre plate on a Roche GS FLX system using Titanium sequencing chemistry, and processed as described below. For the IL assembly experiment, index tagged SC and J5 libraries were pooled and subjected to single end-sequencing in a single channel on the Illumina HiSeq 2000 platform. For the DL assembly experiment, the bulked RNA sample was subjected to single end sequencing in a single channel on the Illumina GAIIx platform. Illumina sequence data cleaned of SMART adapter sequences and with sequencing adapters and terminal N stretches clipped were downloaded from the sequencing provider in FASTQ format and used for this study. Illumina reads were 76nt (GAIIx) or 101nt (HiSEQ 2000) after removal of sequencing adapters. Additional clipping in the cleaning process implemented by the sequencing provider occasionally removed terminal N-stretches from the end of sequences, but this was so infrequent (about 1 in 10,000 reads) that the “mean” read lengths of the cleaned datasets used for the assemblies were effectively still 76 nt (GAIIx reads) or 101 nt (HiSeq 2000).

### De novo sequence assembly of 454 reads and primary characterisation of the assembly products

Fasta format sequences, quality scores and traceinfo-related xml information were parsed into separate files from the *sff* file of the sequence provider, containing 768,512 reads. Vector ends, adapter sequences were stripped from the reads using SSAHA2 (v2.5.3 [24]) and SMALT (v.0.4.1 [25]). Reads were assembled by MIRA (v.3.2.1 [19]) with he *job=denovo,est,normal* parameters on a single node of a High Performance Linux cluster with 72 GB of memory. Contigs were clustered using CD-HIT (v4.54 [20]. Chloroplast specific contigs were filtered out by BLAST-based comparison of individual contigs against the complete chloroplast sequence of *T. subterraneum* (GenBank Accession Nr: EU849487). Non-chloroplast contig sequences were compared to *M. truncatula* transcript sequences using the tblastx program of the BLAST package. Databases were built for standalone searches using the BLAST program [22].

The *M. truncatula* Gene Index, hosted at the Dana Faber Cancer Institute, Boston, USA [26] was used to build a local BLAST database. The latest version (MtGI rel. 10.0, April 15, 2010) available at the time of the analysis contained 68,848 unique sequences.

### Gene ontology and annotation

All protein sequences belonging to *Viridiplantae* were downloaded from GenBank (Release 182.0 [27]). 1,763,836 downloaded protein sequences were clustered by the CD-HIT program at a similarity level of 0.95. The resulting non-redundant sequence set contained 575,865 sequences. These were used to build a stand-alone BLAST database to carry out local BLAST searches. Sequences were subjected to *blastx* searches against the non-redundant protein databases. The *blastx* result files were used for functional annotation and assigning of Gene Ontology (GO) terms via the command line version (B2G4Pipe) of the Blast2GO program [23]. Blastx result files were produced on a local server in automated mode, directed by a shell script and saved by the *-m7* output format option (xml output). Result files were merged in batches of 10,000 files and loaded into the Blast2GO annotation pipeline. An e-value hit prefilter of 1E-6 was applied and an annotation cut-off of 55 and a GO weight of five were set. The primary ontology term annotations were augmented by Second Gene Ontology Layer data through the GO Annotation Toolbox [28] as implemented in the Blast2GO program.

### Mapping assembly of short reads and SNP discovery

Short reads were indexed and aligned onto the S1 reference sequences by the Burrows-Wheeler Aligner (BWA, v0.5.90) [16], using the default parameters for single-end reads. In the case of the inbred line assembly, short reads from two genotypes (J5 and SC) were separately aligned onto the reference sequence backbones and then the alignments were merged using the SAMtools *merge* command. SAM format alignments were indexed and converted to BAM format using SAMtools.

Variant calls from this merged alignment were performed in two subsequent steps:

(1) A global variant call (i.e. including both read types at each variants position) was conducted using the SAMtools *mpileup* command. The resulting pileup file was further processed by the *bcftools* and *vcfutils varFilter.pl* programs of the SAMtools package allowing a maximum read depth of 200. The resulting variant list served for base quality recalibration and local realignment of the combined (J5/SC) BAM file using utilities of the Genome Analysis Toolkit (GATK) package [17].

(2) For SNP discovery, read-type (J5 and SC respectively) specific variants were called from the cleaned J5SC BAM file in two independent steps using the SAMtools *pileup* command.

A similar pipeline was applied for mapping and aligning of short reads from the divergent genotype pool, except conducting of read group related processing steps.

### SNP filtering and classification

Read-type specific raw variant lists were filtered by the SAMtools *varFilter.pl* utility by setting a minimum read depth threshold of six and a maximum read depth threshold of 200. Indel positions were skipped and final SNP calls were acquired by setting a Phred score quality threshold of 20. Using custom Perl scripts, the cleaned pileup files were compared to select variants with matching positions in two compared read groups. From the remaining parts of the pileup file lists, unique variant positions were created. These lists were used to call pileup information from the corresponding BAM format assembly file in regard to the opposing read group using the SAMtools *pileup* program by switching off the variant filtering option. The first eight columns (containing Reference sequence name, Position, Reference base, Read consensus, Consensus quality, SNP quality, Mapping quality and Read coverage respectively) were extracted from the pileup files for each read type and were put into a general matrix containing all relevant variant information. Perl scripts were applied to compare the nucleotide consensus of each read type at each variant position based on intra- and inter-genotype polymorphisms, ambiguity type and relation to the reference in order to sort variants into different categories. Further custom Perl scripts were applied to produce ordered variant lists for each reference sequence and to calculate distributions and frequencies.

### Manual examination for haplotype structure for estimation of heterozygosity

A BAM format alignment file consisting of the reference transcripts and the mapped J5 and SC Illumina reads was visualised in the IGV (v2.0.34 [29]) alignment viewer window. The viewer was initially set to the lowest resolution, in which the read sequences were represented as coloured bars: bases identical to the references sequences were shown as a homogeneous background (different colours for each read types), substitutions were emphasised by base-specific colours and deletions were shown as gaps. Mapped reads were sorted by genotype and surveyed in an approx. 100 bp wide window, sliding along the whole contig length (horizontally), and up- and downwards through the whole alignment (vertically) at positions where two or more variants were indicated within a range of a single Illumina read length (101 nt). After visual scoring the individual reads that covered the same variant positions, the occurrences of the represented haplotypes were recorded. During the process, the following rules were applied: (1) regions with a read depth of less than four were not scored, (2) differing variants of single occurrence were usually not considered, (especially if they were likely the products of sequencing errors, e.g. if they were located at the end of the reads with low base quality scores).

### Haplotype reconstruction

Short reads mapped on the selected contigs from the reference transcript set were extracted – separately for each read type - from the J5SC assembly and re-aligned onto the reference sequences using the FSA alignment program [30]. In each alignment reads were manually ordered into groups at each distinguishing SNP position in a graphical sequence editor window (GDE, [31]). This sorting process resulted in two main groups that contained approximately 99% of the reads, indicating that the reads represented two haplotypes. Reconstructed haplotypes representing consensus sequences and published sub-genomic sequences were aligned with the ClustalW2 program [32]. After cutting-off protruding ends the sequences were re-aligned to generate phylogenetic trees. Graphical trees (unrooted radial phenograms) were drawn by the *Drawtree* program of the PHYLIP package [33].

## Competing interests

The authors declare no competing interests.

## Authors’ contributions

IN participated in planning the study, carried out all of bioinformatics analyses and participated in writing the manuscript. SB participated in the conception, planning and writing of the study, and directed some of the laboratory based experiments. MTA participated in the conception, planning and writing of the study. JMC carried out all in-house plant and laboratory experiments associated with the study and contributed to the writing of the manuscript. DM conceived and planned the study, directed the work, contributed to analyses, and participated in the writing of the manuscript. All authors have read and approved the final version of the manuscript.

## Authors’ information

Teagasc - Crops, Environment and Land Use Programme, Oak Park Research, Carlow, Ireland. Department of Molecular Biology and Genetics, Aarhus University, Forsøgsvej 1, DK-4200 Slagelse, Denmark. Institute of Biological, Environmental & Rural Sciences, Aberystwyth University, Penglais, Aberystwyth, Ceredigion, SY23 3DA , UK. Current address: International Institute of Tropical Agriculture, PMB 5320, Oyo Road, Ibadan, Nigeria.

## Supplementary Material

Additional file 1 Summary table of the GO annotations. Click here for file

Additional file 2 **A multi fasta file containing 71,545 transcript reference sequences.** Contigs are marked with the prefix wcc or wcd, singlets have the prefix wcs. The header of each sequence entry contains the description resulting from the annotation procedure (if any) as well as the number of assigned GO terms and the number of detected SNPs. Click here for file

Additional file 3 **Full list of variants identified in the IL and DL assemblies.** A list of contigs and singletons from the reference transcript assembly with the number, type, position and nucleotide variants of all SNPs identified in the DL and IL assemblies. A full description of the fields in the table is given in the file header. Click here for file

Additional file 4 **Transcript reference sequences with homology to published sub-genomic sequences (from Hand et al. [**[12]**]).** Click here for file

Additional file 5 **Sequence alignments of reconstructed haplotype-specific consensus sequences and published sub-genomic sequences.** Acyl-coA oxidase: The transcript reference sequence wcd-2781 and the reconstructed haplotype-representative consensus sequences (SC_CONS_O, SC_CONS_P’, J5_CONS_O, J5_CONS_P’ were aligned to the published sequences GU443966_9 (ACYL_O) and GU443966_17 (ACYL_P’) respectively. Dehydration responsive element binding protein “DREB3”: The transcript reference sequence wcd-12699 and the reconstructed haplotyp- representative consensus sequences (SC_CONS_O, SC_CONS_P’, J5_CONS_O, J5_CONS_P’) were aligned to the published sequences GU443966_13 (DREB3_O) GU443966_21 (DREB3_P’). Click here for file
