# Supplementary material for: A hybrid next generation transcript sequencing-based approach to identify allelic and homeolog-specific single nucleotide polymorphisms in allotetraploid white clover
Source: BMC Genomics. 2013 Feb 13;14:100. doi: 10.1186/1471-2164-14-100 (PMC3727989; doi:10.1186/1471-2164-14-100)
Supplement: Additional file 4 — Transcript reference sequences with homology to published sub-genomic sequences (from Hand et al. [[12]]). [file 1471-2164-14-100-S4.pdf]

Transcript reference sequences with homology to published sub-genomic sequences (Hand et al. 2010)

Contig ID	Contig lengh, bp	Mean cov. in the IL assembly	Gene product	BAC (O)	BAC (P')	SNPs on the contigs
wcc-13604	1108	32.21	unknown protein	GU443966_11	GU443964_19	IH:2, HE:9, X:3
wcd-2781	2325	186.50	acyl-coA oxidase	GU443966_9	GU443964_17	IH:10, HE:6
wcd-3096	1176	60.75	kruppel-like zinc finger protein ZPT2"	GU443962 (partial)	GU443959_19	S:4
wcd-4226	2104	118.45	translation initiation factor-related	GU443963_7	GU443961	IH:17, HE:22, X:1
wcd-11359	715	18.50	galactose oxidase	GU443962_7	GU443959_15	X:2
wcd-12699	938	71.56	dehydration responsive element binding protein "DREB3"	GU443966_13	GU443964_21	IH:5, HE:8, S:2, X:1
