# Supplementary material for: A hybrid next generation transcript sequencing-based approach to identify allelic and homeolog-specific single nucleotide polymorphisms in allotetraploid white clover
Source: BMC Genomics. 2013 Feb 13;14:100. doi: 10.1186/1471-2164-14-100 (PMC3727989; doi:10.1186/1471-2164-14-100)
Supplement: Additional file 5 — Sequence alignments of reconstructed haplotype-specific consensus sequences and published sub-genomic sequences. Acyl-coA oxidase: The transcript reference sequence wcd-2781 and the reconstructed haplotype-representative consensus sequences (SC_CONS_O, SC_CONS_P’, J5_CONS_O, J5_CONS_P’ were aligned to the published sequences GU443966_9 (ACYL_O) and GU443966_17 (ACYL_P’) respectively. Dehydration responsive element binding protein “DREB3”: The transcript reference sequence wcd-12699 and the reconstructed haplotyp- representative consensus sequences (SC_CONS_O, SC_CONS_P’, J5_CONS_O, J5_CONS_P’) were aligned to the published sequences GU443966_13 (DREB3_O) GU443966_21 (DREB3_P’). [file 1471-2164-14-100-S5.pdf]

dehydration responsive element binding protein "DREB3" GU443966_13 / GU443966_21

```
  wcd-12699    1 AAAACATGTCAACAGCTATAAATANACTACAATAGACAACAAAACATCACATATGTTC---TAGATCCATTTTAATAGAAGAACTAATGAAAGCACTTGA   97
   DREB3_P'    1 -----...................T-.........CA.CA...........C...G.TTCT...........A.T--.......................   92
 SC_CONS_P'    1 ----------------------------------------------------------...----------------.......................   26
 J5_CONS_P'    1 ----------------------------------------------------------...---------------------------------------    3
    DREB3_O    1 -----...................C-.........CA.CA.......T...C...G.TTCT...........A.T--.......................   92
  SC_CONS_O    1 ----------------------------------------------------------...----------------.......................   26
  J5_CONS_O    1 ----------------------------------------------------------...---------------------------------------    3

  wcd-12699   98 GCCTTTTATCAAAAGTGATTATTCAACAATCTCTTCTTCCACACATGAACAATCAAACTTTCCCTCAACTTCTTATTCACCATTTTCACCCAATTACTTC  197
   DREB3_P'   93 ......C................................T...............G...................C........................  192
 SC_CONS_P'   27 ......C................................T...............G...................C........................  126
 J5_CONS_P'    4 -------................................T...............G...................C........................   96
    DREB3_O   93 .......................................T...............C............................................  192
  SC_CONS_O   27 ....................................................................................................  126
  J5_CONS_O    4 -------.............................................................................................   96

  wcd-12699  198 ACCTACGAAACCCAAACAAGCTCAATAGGACTCAACCAACTCACYCCATCACAAATCCTTCAAATCCAAACCCAAATTCAAATTCCCCAAAAAAGCCATC  297
   DREB3_P'  193 ............................................C.......................................................  292
 SC_CONS_P'  127 ............................................C.......................................................  226
 J5_CONS_P'   97 ............................................C.......................................................  196
    DREB3_O  193 ............................................C.......................................................  292
  SC_CONS_O  127 ............................................T.......................................................  226
  J5_CONS_O   97 ............................................C.......................................................  196

  wcd-12699  298 TTTCTCAAAAGCCCGTTTCAATGAAGCACTTTTCTACTCCTTCAAAACCAATGAAACTTTACCGTGGAGTTAGACAACGTCATTGGGGAAAAATGGGTCG  397
   DREB3_P'  293 ...................C..............G........G.....G..A.......................................-.....T.  391
 SC_CONS_P'  227 ...................C........................................................................-.......  325
 J5_CONS_P'  197 ............................................................................................-.......  295
    DREB3_O  293 ....C..............C.............................T........C.................................-.......  391
  SC_CONS_O  227 ............................................................................................-.......  325
  J5_CONS_O  197 ............................................................................................-.......  295

  wcd-12699  398 CTGAAATTAGACTCCCAAGAAACCGTACTCGTTTATGGTTAGGTACATTTGAAACTGCAGAGGAAGCAGCTTTTGCTTATGATAACGCAGCTTAWTAAGC  497
   DREB3_P'  392 .......A........................C.C...........................................................-.....  490
 SC_CONS_P'  326 ................................C.C...........................................................-.....  424
 J5_CONS_P'  296 ..............................................................................................-.....  394
    DREB3_O  392 ..............................................................................................-.....  490
  SC_CONS_O  326 ..............................................................................................-.....  424
  J5_CONS_O  296 ...................................................................G..........................-.....  394

  wcd-12699  498 TTAGAGGTGAATTTGCACGACTCAATTTTCCTCACATGAGTCACCATGAAAATTACAATCCTCTCCCTTCTTCTGTTGATTCAAAGCTTCAAGCAATTTG  597
   DREB3_P'  491 ......................T.......................................................................T.....  590
 SC_CONS_P'  425 .......................C..........T...T............................A..........................T.....  524
 J5_CONS_P'  395 ..................................T...T............................A..........................T.....  494
    DREB3_O  491 ....................................................................................................  590
  SC_CONS_O  425 ....................................................................................................  524
  J5_CONS_O  395 ....................................................................................................  494

  wcd-12699  598 TGAAACTTTAGCCATTTCTCAAAAACAGGGGAAACAGGGGAACAATGAAAAACCATGTTCCTCTGTTGTTGAAGATGTAAAACCGGTTGTTTGTGTTACT  697
   DREB3_P'  591 ....................................................................................T.............G.  690
 SC_CONS_P'  525 .....................................................TC.............................A.............G.  624
 J5_CONS_P'  495 .....................................................TC.............................A.............G.  594
    DREB3_O  591 ....................................................................................................  690
  SC_CONS_O  525 ....................................................................................................  624
  J5_CONS_O  495 .A.........................................G.......................C................................  594

  wcd-12699  698 GAAAAACCGAAGGATGAATTGGTTTGTCACAAGTTTGAGGATTTTAAGGTTGAGAATGAGAAGACAACGATGTCATCTATGTCTGATGAATGTTCCCCTT  797
   DREB3_P'  691 ........A..................................................A.......T......G.G..................A....  790
 SC_CONS_P'  625 ........A..................A...............................A.......T......G....................A....  724
 J5_CONS_P'  595 ........A..................A...............................A.......T.....TG....................A....  694
    DREB3_O  691 ...........................T........................................................................  790
  SC_CONS_O  625 ....................................................................................................  724
  J5_CONS_O  595 .C......A..A...............................................A.......T......G....................A....  694

  wcd-12699  798 GTTTGTCATCACCAGAATCTGGTGTTACTTTCTTGGATTTCTCAGATTCTAGCCAGTGGGATAAAGTGGAGAATTTTGGGTTGAAGAAGTACCCTTCTGT  897
   DREB3_P'  791 ..........T............................................T......G.....................................  890
 SC_CONS_P'  725 ..........T............................................T......G....................................-  823
 J5_CONS_P'  695 ..............................................................G.....................................  794
    DREB3_O  791 ....................................................................................................  890
  SC_CONS_O  725 ....................................................................................................  824
  J5_CONS_O  695 ..........T............................................T......G.....................................  794

  wcd-12699  898 TGAAATTGATTGGGAAGCTTTATGAGTCTTTGAAGTGATTT                                                              938
   DREB3_P'  891 .........................----------------                                                              915
 SC_CONS_P'  824 -----------------------------------------                                                              823
 J5_CONS_P'  795 ..................................-------                                                              828
    DREB3_O  891 .........................----------------                                                              915
  SC_CONS_O  825 .........................................                                                              865
  J5_CONS_O  795 ......................................---                                                              832
```

acyl-coA oxidase GU443966_9 /GU443966_17

```
   wcd-2781 GGGTTCTTTGATCAGATGCAACCACTATTATACTCCAATTTCAACAATGAGCTAAAATCACTTTTCATATTGATTCCTCCAACTAAATATTCATCTTCTT   100
    ACYL_P' ----------------------------------------------------------------------------------------------------     0
 SC_CONS_P' -------------......................................G.............A...........................---....    84
   J5_CON_P' -----------------...................................................................................    83
     ACYL_O ----------------------------------------------------------------------------------------------------     0
  SC_CONS_O -------------......................................G.............A...........................---....    84
    J5_CON_O -----------------...................................................................................    83

   wcd-2781 TGGGGACCTTAATTTATTCGAATCCGACATATTGTGTGTACTGAGAGTAAGCTCCTGATTGCTTCGCAATAGGTGCCCGTGTTACATGATCTGGAAGATC   200
    ACYL_P' -------------......A................................................................................    87
 SC_CONS_P' ...................A................................................................................   184
   J5_CON_P' ...................A................................................................................   183
     ACYL_O -------------.......................................................................................    87
  SC_CONS_O ....................................................................................................   184
    J5_CON_O ....................................................................................................   183

   wcd-2781 AAATGCATCAACAAGCTCCTTTGCAATATTCCTCACTTGGAAACTTAGATACTCTGACAATTTGTGGATTGCCTTGGCTTTGTTGGGAGCCACATAGTCA   300
    ACYL_P' ......G.............................................................................................   187
 SC_CONS_P' ......G.............................................................................................   284
   J5_CON_P' ......G.............................................................................................   283
     ACYL_O ....................................................................................................   187
  SC_CONS_O ....................................................................................................   284
    J5_CON_O ....................................................................................................   283

   wcd-2781 ACATTGCGGTAGGTTCCAATGTCGTTCCATATTCTATCCAGAGCATAAAGATCACAGACAAGCTTCAAAGCAGCTTGAGCGCTTGGATCAGGACAGCTCT   400
    ACYL_P' ..................................G.................................................................   287
 SC_CONS_P' ..................................G.................................................................   384
   J5_CON_P' ..................................G.................................................................   383
     ACYL_O ..................................G.................................................................   287
  SC_CONS_O .................................................................................................---   381
    J5_CON_O ..................................G.................................................................   383

   wcd-2781 GCACAGCTTCAATGAATTTAGCAAGAATGACCGACTCAATATGTGATTCTGCAAGTGTCAATAGGTGATTTAAGCATCTATTCCATGCACCAAACTGCCC   500
    ACYL_P' .T.............................T....................................................................   387
 SC_CONS_P' .T.............................T....................................................................   484
   J5_CON_P' .T.............................T....................................................................   483
     ACYL_O ....................................................................................................   387
  SC_CONS_O -...................................................................................................   480
    J5_CON_O ....................................................................................................   483

   wcd-2781 TAGAGGTTTTGAGTGCTTTTGAAGTCGTACAGCAACACTATGAAGTAATCTAGACGTTCGGTATCTGAAAGCATCCAATTGAAATTTAGGATCTCGTAGA   600
    ACYL_P' ...............................................................-------------------------------------   450
 SC_CONS_P' ...............................................................-------------------------------------   547
   J5_CON_P' ...............................................................-------------------------------------   546
     ACYL_O ..................C...................................T.............................................   487
  SC_CONS_O ....................................................................................................   580
    J5_CON_O ...............................................................................................T....   583

   wcd-2781 TGGTCTCCGCCTTCCCACCTAGCAGTAACCGGGTTTGGTTGTGACAGATAAGTATTCATGGAATCTCTTAAGTAGTTCCATGTTGCTGCTAAAGTCCCTC   700
    ACYL_P' ----------------------------------------------------------------------------------------------------   450
 SC_CONS_P' ----------------------------------------------------------------------------------------------------   547
   J5_CON_P' ----------------------------------------------------------------------------------------------------   546
     ACYL_O ....................................................................................................   587
  SC_CONS_O ....................................................................................................   680
    J5_CON_O ....................................................................................................   683

   wcd-2781 CTTTGAACTTTCTCTGGTATTGTTTCAAAAGATCGGCTGCAACCTGTTGAAGAAGAACCGTGTTGTCTCCTTCAAATGTCTGGAAAATGTCATGATCATT   800
    ACYL_P' -------------------------------------------......................................................G..   507
 SC_CONS_P' -------------------------------------------........................................................C   604
   J5_CON_P' -------------------------------------------.........................................................   603
     ACYL_O ....................................................................................................   687
  SC_CONS_O .......................................S...........................................................C   780
    J5_CON_O ....................................................................................................   783

   wcd-2781 CCTCAAGGAACCAAACCGATTTACAGCAGCATAACCATGTCCTCCACAGGCTTCCCTACAGACGCTGAGTGATTTTGCGGTATATGATGTTACATAAGCC   900
    ACYL_P' ..............................................................T.....................................   607
 SC_CONS_P' ...A..........................................................T.....................................   704
   J5_CON_P' ..............................................................T.....................................   703
     ACYL_O   ....................................................................................................   787
  SC_CONS_O ...A................................................................................................   880
    J5_CON_O ..............................................................T.....................................   883

   wcd-2781 TTCAGACCTGCCGAGAGAGCATGAACATCTGCAACTAATTCTTCATCGTGAGACTTTTTCATTTGAGCGTATTTCTCCACTAAGTTTGTAGTGGCAAAAT  1000
    ACYL_P' ...............................................A........C..............C............................   707
 SC_CONS_P' ...............................................A........C...........................................   804
   J5_CON_P' ...............................................A........C...........................................   803
     ACYL_O ......................................................A.............................................   887
  SC_CONS_O ....................................................................................................   980
    J5_CON_O ....................................................................................................   983

   wcd-2781 GAAATGCATAAGTTGAAGCCAGCATTGGCATAAGCTTATGCTGATGAGACTGGTAGTCGAGAATGGTGACTTCGGGTTGGTTTGGAGGTCCAAATTGCTG  1100
    ACYL_P' ..................................T........G........A....................A..........................   807
 SC_CONS_P' ..................................T.................................................................   904
   J5_CON_P' ..................................T.................A...............................................   903
     ACYL_O   ....................................................................................................   987
  SC_CONS_O ....................................................................................................  1080
    J5_CON_O ....................................................................................................  1083

   wcd-2781 ACGAATTAGAGAATATCTGATGGCGATTGTGGAAGCAACCTTGAGGACACTAACAGAAGAATATGCAAGGCCTACCCTACCACCAACGAGTTCTCCAAGA  1200
    ACYL_P' ...............C........A.......C............................................................C......   907
 SC_CONS_P' ...G....................A.......C............................................................C......  1004
   J5_CON_P' ........................A.......C............................................................C......  1003
     ACYL_O ....................................................................................................  1087
  SC_CONS_O ....................................................................................................  1180
    J5_CON_O ........................A...........................................................................  1183

   wcd-2781 GTTGCACCAAACCGCTTATTAACCGAAGGAAGGGTACTTGTGTACTGGCCATCACGGGAAACATCTCCAAAACGGTTTAGAAGATTATCTCGAGGAATTC  1300
    ACYL_P' ....................................................................................................  1007
 SC_CONS_P' ....................................................................................................  1104
   J5_CON_P' ............................-.......................................................................  1102
     ACYL_O ....................................................................................................  1187
  SC_CONS_O ....................................................................................................  1280
    J5_CON_O ............................-.......................................................................  1282
```

```
   wcd-2781 TCACAGAGCGGAATC-TCAGAGCTCCATTATCAACGCCATTAAGGCCAACTTTATGACCACAATCATGTATCTCAATTCCAGGAAGTGTTTGATGAGTCT  1399
    ACYL_P' ....................................................................................................  1107
 SC_CONS_P' ....................................................................................................  1204
  J5_CON_P' ...............T....................................................................................  1202
     ACYL_O .................T..................................................................................  1287
  SC_CONS_O ....................................................................................................  1380
   J5_CON_O ...............T....................................................................................  1382

   wcd-2781 TCATATCCCTTATTGGAACAATGAAAGCATGAACACCCATATCAGAAATTCCTTTTGCATCATAATTAGGTAACTTCAACCTAGCAAAAACAGTAGCAAA  1499
    ACYL_P' ....................................................................................................  1207
 SC_CONS_P' ....................................................................................................  1304
  J5_CON_P' ....................................................................................................  1302
     ACYL_O ....G...............................................................................................  1387
  SC_CONS_O ....................................................................................................  1480
   J5_CON_O ....................................................................................................  1482

   wcd-2781 CTTTCCATGCACCGCGGCATTGCCAATCCACCATTTGATGGCACCGTCATTCGGAGTATCAACAATAAATTCATCAGTGATCGGATCAAAGGTGGCAACA  1599
    ACYL_P' ...G................................................................................................  1307
 SC_CONS_P' ........C.C.........................................................................................  1404
  J5_CON_P' ....................................................................................................  1402
     ACYL_O ...G.................T.....................................................G........................  1487
  SC_CONS_O ........C.C.........................................................................................  1580
   J5_CON_O ....................................................................................................  1582

   wcd-2781 GTTTGAAGGCCTTGCACATTTGAACCATGGTGAAGCTCAGTCATAGCAAAACAACCAGGAAAATCCAAGTTATCAATACCATCATAGTACTTATCCTTGT  1699
    ACYL_P' ..........................................................................C.........................  1407
 SC_CONS_P' ....................................................................------..........................  1498
  J5_CON_P' ....................................................................................................  1502
     ACYL_O ...................................................................................G................  1587
  SC_CONS_O ....................................................................------..........................  1674
   J5_CON_O ...................................................................................G................  1682

   wcd-2781 GTTTCTGTGTTCCCAAATTGATAACAGAACCACCCCAAAGACTATATTGAACACCCATTTTGATTCCAAGCGACATGTCAACACTACCAACAGCTTCCAA  1799
    ACYL_P' ............................................................................A.......................  1507
 SC_CONS_P' ....................................................................................................  1598
  J5_CON_P' ....................................................................................................  1602
     ACYL_O ......................................................................T.....A.......................  1687
  SC_CONS_O ....................................................................................................  1774
   J5_CON_O ....................................................................................................  1782

   wcd-2781 AATAGCAAAATACTTAGCAGGATCATGAACAACATAATGAAGAGGTCTAATTCCAGCTTCTCTAACAAGACCCAAAAGCTGATTCATACATAATTCTCTA  1899
    ACYL_P' ....................................................................................................  1607
 SC_CONS_P' ....................................................................................................  1698
  J5_CON_P' ....................................................................................................  1702
     ACYL_O ....................................................................................................  1787
  SC_CONS_O ....................................................................................................  1874
   J5_CON_O ....................................................................................................  1882

   wcd-2781 TGATTATCTTTRGAAATTTCAAGAGGGGTTTGAAGTTGAGGATTAGCGTTGAAATAATCAAAAACTTTYTCTTGTATATCTCTGTATTTTCCTCTCATGT  1999
    ACYL_P' ...........A........................................................C..............A................  1707
 SC_CONS_P' ...........A........................................................T..............A................  1798
  J5_CON_P' ...........A........................................................T..............A................  1802
     ACYL_O ...........A........................................................C..............A..............--  1885
  SC_CONS_O ...........G........................................................C.............................--  1972
   J5_CON_O ...........A........................................................C...............................  1982

   wcd-2781 AACTTGTTAAAGAATTCGCATCGACAATTAACTTCTTACCACGACTGCAATTTTCCATCTCTAATGGATGATGATTCCATGTGAGACGAGGAATTGGGTT  2099
    ACYL_P' ...C..................................................................................TT.A..........  1807
 SC_CONS_P' ....................................................................................................  1898
  J5_CON_P' ...C..............................T...................................................TT.A..........  1902
     ACYL_O ----------------------------------------------------------------------------------------------------  1885
  SC_CONS_O ----------------------------------------------------------------------------------------------------  1972
   J5_CON_O ..................................T...................................................TT.A..........  2082

   wcd-2781 TAGGTGTAAGGATAGTCTTTGGATTCGTCTCTCTGCTTCGGAATTTGATTCGTTGTTAGATTTTAGGGTTTGCATTTTGTGTGTGTGTGTTTGTTTGGGA  2199
    ACYL_P' ..............A.....C..............................A.......................-------------------------  1882
 SC_CONS_P' ..............A.....................................................................................  1998
  J5_CON_P' ..............A.....C..............................A.......................-------------------------  1977
     ACYL_O ----------------------------------------------------------------------------------------------------  1885
  SC_CONS_O ----------------------------------------------------------------------------------------------------  1972
   J5_CON_O ..............A.....C..............................A.......................-------------------------  2157

   wcd-2781 TTGAAGAAATGAAATTAGGGGAGAATGATGGAGGAATGAGGAAGTTGAATGTTGAATGATATAGTCCCCGCGTACTCTGCGTTGATACCACTGCTTAAGC  2299
    ACYL_P' ----------------------------------------------------------------------------------------------------  1882
 SC_CONS_P' .........-------------------------------------------------------------------------------------------  2007
  J5_CON_P' ----------------------------------------------------------------------------------------------------  1977
     ACYL_O ----------------------------------------------------------------------------------------------------  1885
  SC_CONS_O ----------------------------------------------------------------------------------------------------  1972
   J5_CON_O ----------------------------------------------------------------------------------------------------  2157

   wcd-2781 AGTGGTATCAACGCAGAGTACTTTTT                                                                            2325
    ACYL_P' --------------------------                                                                            1882
 SC_CONS_P' --------------------------                                                                            2007
  J5_CON_P' --------------------------                                                                            1977
     ACYL_O --------------------------                                                                            1885
  SC_CONS_O --------------------------                                                                            1972
   J5_CON_O --------------------------                                                                            2157
```
